# Deletion of Pr72 causes cardiac developmental defects in Zebrafish

**DOI:** 10.1371/journal.pone.0206883

**Published:** 2018-11-27

**Authors:** Guibo Song, Mingjun Han, Zuhua Li, Xuedong Gan, Xiaowen Chen, Jie Yang, Sufang Dong, Ming Yan, Jun Wan, Yanggan Wang, Zhuliang Huang, Zhan Yin, Fang Zheng

**Affiliations:** 1 Center for Gene Diagnosis, Zhongnan Hospital of Wuhan University, Wuhan, China; 2 Department of Cardiology, Zhongnan Hospital of Wuhan University, Wuhan, China; 3 Key Laboratory of Aquatic Biodiversity and Conservation of Chinese Academy of Sciences, Institute of Hydrobiology, Chinese Academy of Sciences, Wuhan, China; 4 Department of Cardiology, Renmin Hospital of Wuhan University, Wuhan, China; Medical College of Wisconsin, UNITED STATES

## Abstract

The alpha regulator subunit B'' of protein phosphatase 2 (PPP2R3A), a regulatory subunit of protein phosphatase 2A (PP2A), was reported to present a special subcellular localization in cardiomyocytes and elevate in non-ischemia failing hearts. *PPP2R3A* has two transcriptions PR72 and PR130. PR72 acts as a negative regulator of the Wnt signaling cascade, while the Wnt signaling cascade plays a pivotal role in cardiac development. And PR130 was found to be involved in cardiac development of zebrafish in our previous study. Thus, to investigate the function of PR72 in heart, two stable *pr72* knockout (KO) zebrafish lines were generated using Transcription Activator-Like Effector Nuclease (TALEN) technology. Homozygous *pr72* KO fish struggled to survive to adulthood and exhibited cardiac developmental defects, including enlarged ventricular chambers, reduced cardiomyocytes and decreased cardiac function. And the defective sarcomere ultrastructure that affected mitochondria, I bands, Z lines, and intercalated disks was also observed. Furthermore, the abnormal heart looping was detected in mutants which could be rescued by injection with wild type *pr72* mRNA. Additionally, it was found that Wnt effectors were elevated in mutants. Those indicated that deletion of *pr72* in zebrafish interrupted cardiac development, probably through activation of the Wnt pathway.

## Introduction

Dysregulation of protein phosphorylation is an important molecular mechanism for numerous cardiac diseases, including heart failure and cardiomyopathy [1, 2]. Recently in addition to kinases, people paid more and more attention to levels and activities of protein phosphatases in the process of heart diseases [2, 3]. Protein phosphatase 2A (PP2A), one of the major serine/threonine phosphatases, is composed of three subunits: scaffold A subunits, catalytic C subunits and variable regulatory B subunits [4, 5]. There are at least four families of PP2A regulatory B subunits, and each family has more than one familiar members: identified as B (PPP2R2A/B/C/D), B' (PPP2R5A/B/C/D/E), B'' (PPP2R3A/B/C) and B‴ (PPP2R4) [2]. The activity, specificity and subcellular localization of the PP2A heterotrimeric holoenzyme complex, are highly regulated through the interaction of regulatory B subunit family members with the substrates [6]. PP2A contributes to a central role in cardiac diseases [7–9]. Transgenic mouse lines expressing either mutated structural A subunits or catalytic C subunits exhibit a cardiac phenotype resembling dilated cardiomyopathy [10, 11]. Deletion of the regulatory B56γ subunit, results in heart development defects, including reduced cardiomyocytes (CMs) and a ventricular septal defect [12]. However, depletion of B56α results in improved cardiac function [13]. So far, the role and regulation of this critical enzyme family especially subunit B, was still largely elusive in cardiac diseases.

PPP2R3A (PP2A, regulatory subunit B'', alpha, NM_181897.2), as one member of regulatory subunit B'' family, was found to be located on both Z- and M-lines in cardiomyocytes and has the differential transcriptional regulation in heart failure [2]. PR72 and PR130 are two different transcriptions of *PPP2R3A*. PR72 has an N terminus that differs from that of PR130 but shares the same C terminus. PR72 is a negative regulator of Wnt signaling pathway, while Wnt pathway plays an important role in heart development [14]. In our previous study, we found that PR130 was required for normal cardiac development [15]. Thus, we made a hypothesis that PR72 might also play a major role in heart development.

To investigate the function of PR72 in heart, we created two *pr72* knockout (KO) zebrafish lines, and found that *pr72* KO zebrafish showed cardiac defects, which indicating that PR72 might involve in cardiac development.

## Materials and methods

### Zebrafish lines

The AB zebrafish and cmlc2: EGFP transgenic zebrafish were maintained as previously described [16]. Briefly, zebrafish were maintained at 28.5°C with a 14-hour-light/10-hour-dark photoperiod and fed brine shrimp three times a day. All procedures relating to the care and use of fish were approved by the ethics committee from Institute of Hydrobiology, Chinese Academy of Sciences (Approval Protocol No. IHB2013724). The investigation conforms to the Guide for the Care and Use of Laboratory Animals published by the US National Institutes of Health.

### Whole-mount in situ hybridization

Whole-mount in situ hybridization (WISH) was executed using antisense probes (Primer sequences were presented in S1 Table) for *pr72* and *cmlc2*, which was synthesized using T7 RNA polymerases and labeled with digoxigenin-UTP (Roche, Mannheim, Germany) [16].

### Pr72 Knockout by Transcription Activator-Like Effector Nuclease

We designed Transcription Activator-Like Effector Nuclease (TALEN) arms aimed at the 1st exon of the *pr72* gene to induce somatic mutations. The TALEN target site for *pr72* was designed using the web-tool TALEN-NT, and the TALEN recognition sequences were the left TALEN 5'-GCGGGGTGAACTGGCCT-3' and the right TALEN 5'-GGAAGGACAAAGTCAC-3' [17]. A 15-bp spacer with a SacII sequence was located between the two binding sites. TALEN expression vectors harboring a wild type FokI nuclease were constructed via the Golden Gate Assembly method, linearized by SacI and used as templates for TALEN mRNA synthesis using mMessage mMachine T3 Kit (Ambion) [18]. Equal amounts of left and right TALEN mRNA (100ng/uL) were injected together into 1-cell-stage zebrafish embryos using a Harvard micro-injector (Harvard Apparatus, Holliston, MA, USA). To analyze the induced somatic small indels, groups of embryos were collected at 48 hours post-fertilization (hpf). Deletion events within the target site were screened by PCR-restriction fragment length polymorphism (PCR-RFLP) and confirmed by clone sequencing and western blot. The positive founders were outcrossed with Wt zebrafish, and F1 embryos at 24 hpf were again screened for germ line transmission by PCR-RFLP. Then, the F1 embryos of the positive founders were raised to adulthood, and the tail fins of the adult F1 zebrafish were analyzed individually. The paired positive F1 zebrafish (carrying mutation M1 or M2) were intercrossed to obtain F2 homozygous mutant offspring and wild type (Wt) offspring; the latter were used as the controls (Wt offspring) for the whole study.

### Histological studies

Adult Wt and mutant zebrafish of the same age and similar sizes were bathed in the tricaine buffer (7.65 mmol/L) for 5 min. The hearts were then removed, embedded in Optimal Cutting Temperature (OCT) and stored at -20°C. Fish heart sections (10-μm-thick) were stained with hematoxylin and eosin (HE) before being observed under an Olympus microscope. Morphometric analysis of HE-stained tissues was performed using ImagePro Plus software [19]. The chamber size was compared based on the ratio of the ventricular chamber area to the ventricular area at a magnification of 40×. Myocardium amounts were counted in each of 10 randomly chosen fields per fish at a magnification of 1000× by two observers blinded to the identification of the fish from which the images were obtained.

### Quantification of the heart function

The heart function indices including heart rate, end-diastolic diameter, end-systolic diameter, and fractional shortening, were quantified using videos of beating hearts from transparent 54 hours hpf embryos. The transparent hearts were imaged by differential inference contrast (DIC) imaging, using an IX71 microscope (Olympus) with Optical Heartbeat Analysis software.

### Rescue experiments

To validate the specificity of the *pr72* KO, rescue experiments were performed by injecting 150 pg of Wt *pr72* mRNA into 1-cell-stage zebrafish embryos from Wt and mutant homozygous F2 offspring. The Wt *pr72* cDNA of zebrafish was transcribed from the extracted zebrafish mRNA by reverse transcription PCR (RT-PCR), and subcloned into the pSP64 plasmid expression vector (Primer sequences were presented in S1 Table). Then *pr72* mRNA was synthesized in vitro using mMessage mMachine SP6 Kit (Ambion, AM1340). Heart looping defects were assessed using 48-hpf zebrafish embryos by WISH.

### Transmission electron microscopy

Five adult Wt hearts and nine adult mutant hearts were removed, pre-fixed in 2% glutaraldehyde before being washed three times, for 30 min each, in 0.1 mol/L phosphate buffer saline (PBS), post-fixed in 1% aqueous OsO_4_ for 2 h, and then washed again. The samples were dehydrated in graded ethanol before being dehydrated in acetone and then embedded in Epon 812. Then, 100-nm-thick sections were cut using UC7 ultramicrotome (Leica), stained with uranyl acetate and lead citrate, and imaged under a Hitachi 7700 transmission electron microscope.

### Reverse transcription quantification PCR

Total RNA of embryos and heart tissues was extracted with Trizol (Invitrogen). The time course expression of *pr72* in the embryos at 0, 6, 12, 24, 48, 72 hpf, as well as in the adult hearts was detected by reverse transcription PCR (RT-PCR) and real-time quantification PCR (qPCR). The expression of *gata4* (NM_002052.3), *gata5* (NM_080473.4), *gata6* (NM_131557.1), *nkx2*.*5* (NM_131421.1), *nkd2a* (NM_001098197.1), *β-catenin* (NM_181601.4) were compared between Wt and mutant zebrafish 72-hpf embryos, and the *β-actin* was used as a reference to normalize the results of fish (Primer sequences were presented in S1 Table). The qPCR was executed on a CFX 96 Real time system (Bio-Rad) using SYBR _Green PCR Master Mix (Applied Biosystem). The 2^-Δ^ Cq was used as the comparative expression level by comparative threshold cycle (Cq) method, and Δ Cq was the difference in the threshold cycles for the targeted and reference gene. The experiments were assayed in triplicate.

### Western blot

The body tissue of twenty zebrafish embryos was homogenized using a chilled homogenizer in RIPA buffer. After quantification, tissue lysates were analyzed on Mini-Protein tetra cell (Bio-Rad) using 10% sodium dodecyl sulfate polyacrylamide gel electrophoresis (SDS-PAGE) in Tris/glycine/SDS buffer. Samples were denatured at 100°C for 5 min before loading. Gels were transferred to a PVDF membrane using the Mini-Protein tetra cell in Tris/glycine buffer with 20% methanol (v/v). Membranes were blocked for 2 h at room temperature using a 5% BSA solution and incubated with primary antibody (anti-PR72, Abcam, ab126195) overnight at 4°C.

### Statistics

The normally distributed data shown in the graph are presented as the mean ± SEM. Statistical analyses were performed by one-way ANOVA followed by Dunnett’s posttest, unpaired student’s t test and nonparametric test as indicated in the figure legends, using SPSS 18.0. All *p* values were two-sided. A value of *p* < 0.05 was considered significant.

## Results

### KO of *pr72* induced cardiac phenotype

**Fig 1:**
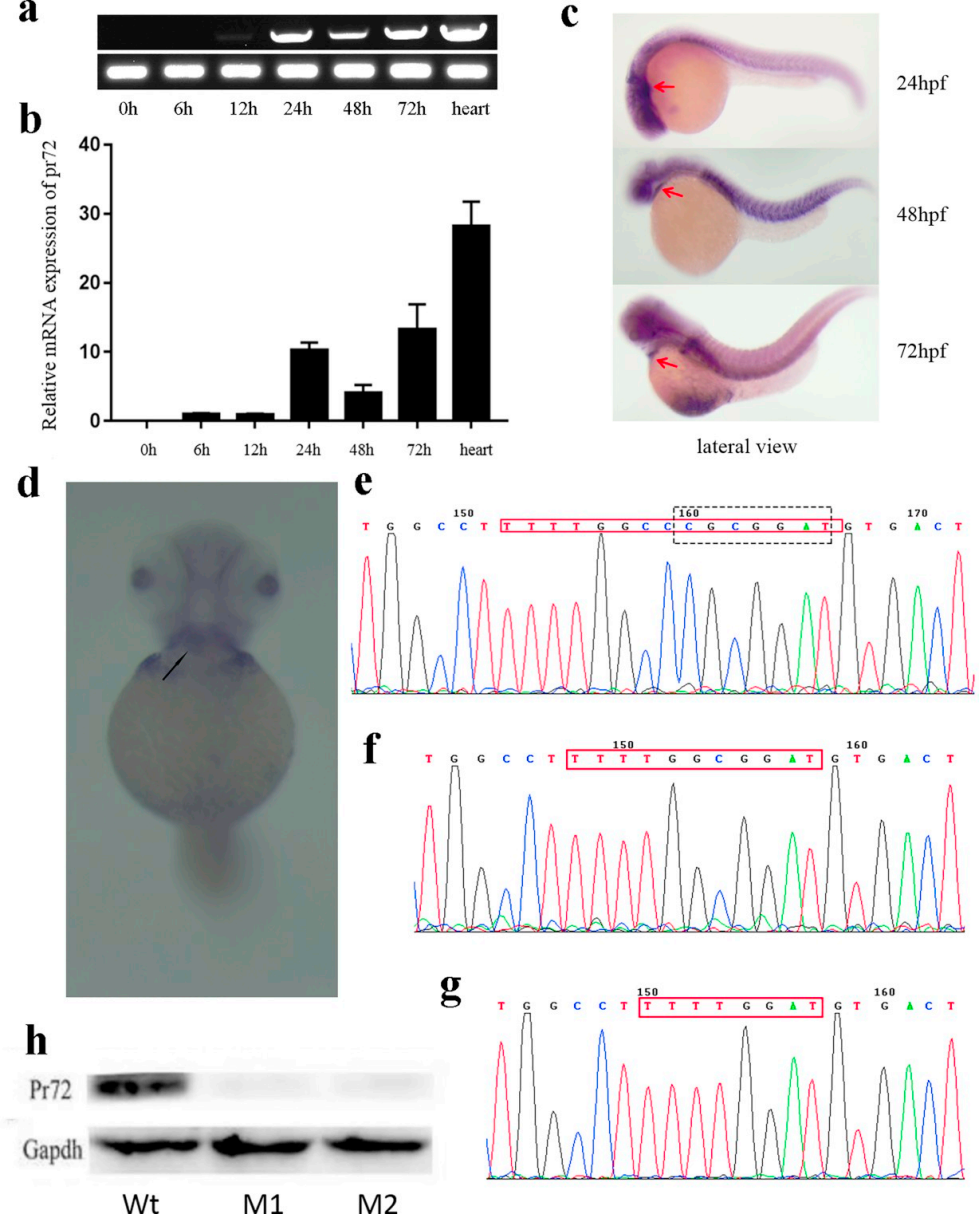
Expression patterns of *pr72* and verification of *pr72* knockout. (a, b) Time course expression of *pr72* in zebrafish embryos at 0, 6, 12, 24, 48, 72 hpf and in adult hearts, identified by RT-PCR (a) and qPCR (b). (c) Expression of *pr72* at 24, 48 and 72 hpf, as shown by whole mount in situ hybridization. A lateral view, anterior to the left, the expression in the heart is shown by red arrows. (d) A ventral view of the in situ result of embryos at 72 hpf, anterior to the top, the expression in the heart is indicated by a black arrow. (e, f & g) The sequencing results of TALEN target sites (indicated by the red frame) in PCR products amplified from F2 zebrafish (M1 (f) and M2 (g)) and the wild type (Wt); the restriction enzyme ScaII recognition site was shown using a black box in Wt. (h) The Pr72 Western-blot results of Wt, M1 and M2.

**Fig 2:**
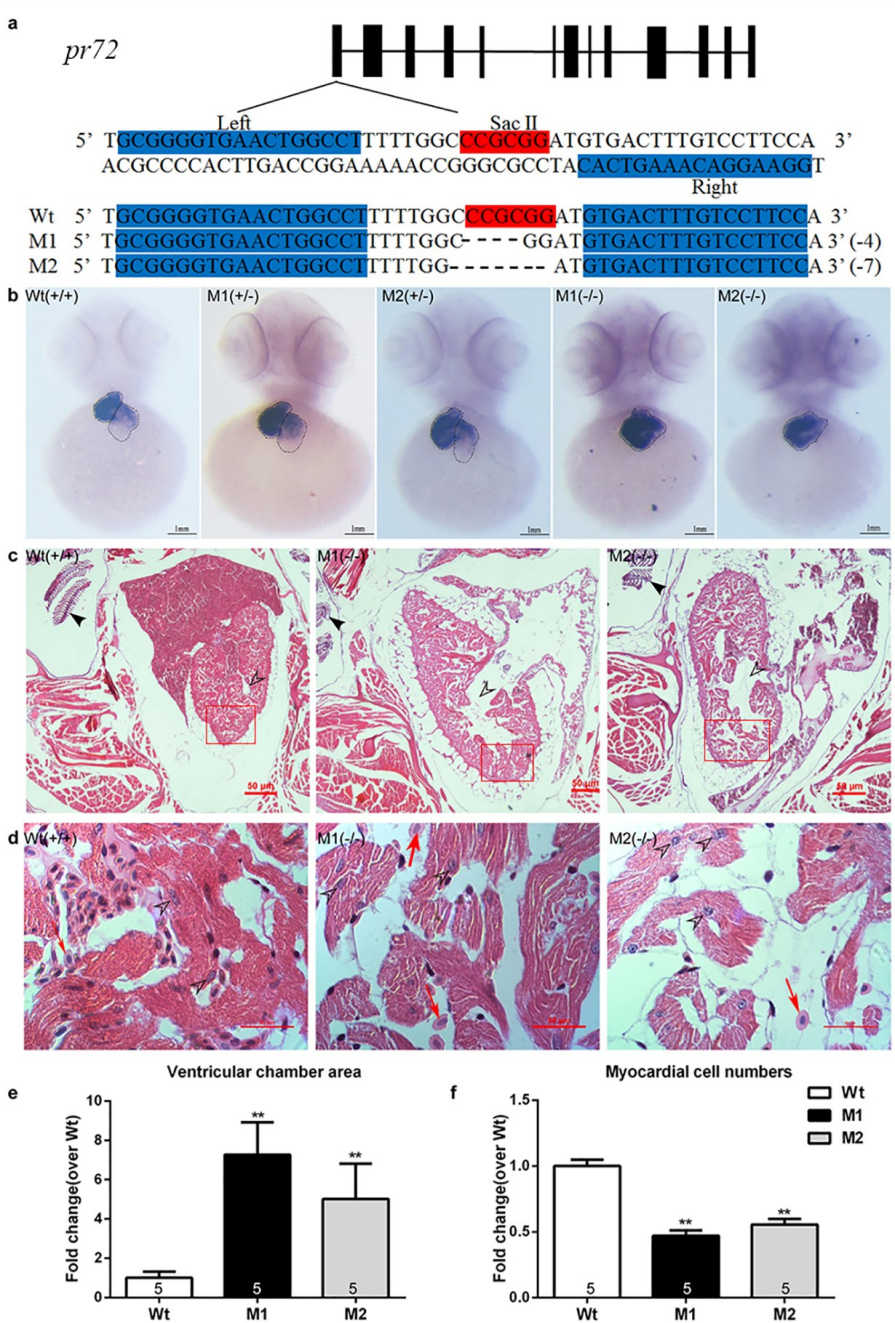
*Pr72* KO mediated by TALEN induced cardiac phenotypes in zebrafish. (a) The TALEN target site of exon 1 of the zebrafish *pr72* gene. The binding sites used in this study (indicated by “Left” and “Right”) are highlighted in cyan; the SacII site in the spacer is highlighted in red. Representative sequencing results revealed different InDels in the TALEN target site. (b) WISH of *cmlc2* expression shows the ventricles and atria in zebrafish embryos, and the ventricles and atria were circled by a dotted line while the ventricle was on the left and the atrium was on the right. The ventricles were enlarged, while the atrial wall was thinning and the atrium was even invisible in the homozygous fish compared to the heterozygous and Wt fish. (c, d) Representative histopathologic sections stained with HE at 40×(c) and 1000×(d), Scale bars 50μM. (c) Representative Hollow arrowheads indicated the ventricular chambers. The bony landmarks pointed by solid arrowheads indicated that the slices were from the same region of the fish body. (d) Higher magnification regions in the red box (1000×). The heart tissues Wt, M1 and M2 were at the same section of the heart. Hollow arrowheads pointed the myocardium nucleus. Red arrows indicated the erythrocytes. (e) Quantification of ventricular chamber areas normalized to ventricular areas, and compared to Wt. (f) Quantification of myocardium cell numbers compared to the control. Myocardium amounts were counted in each of 10 randomly chosen fields per fish at a magnification of 1000× by two observers blinded to the identification of the fish from which the images were obtained. Data are expressed as mean ± SEM. **, *p* < 0.01 vs controls, one-way ANOVA followed by Dunnett’s posttest. The numbers of zebrafish are indicated in the columns.

*Pr72* could be overtly detected in the bodies of 24-hpf, 48-hpf and 72-hpf embryos, as well as in adult hearts (Fig 1). In 24-hpf, 48-hpf and 72-hpf embryos, *pr72* expression was predominantly noted in the heart and somites (Fig 1). These expressional analyses support a function for *pr72* in heart. Two stable deletion mutations were produced using TALEN, and were detected by PCR-RFLP and were validated by DNA sequencing on the fragment containing TALEN target site (Figs 1 and 2), and the western blotting result which showing no Pr72 protein in the mutants (Fig 1).

**Fig 3:**
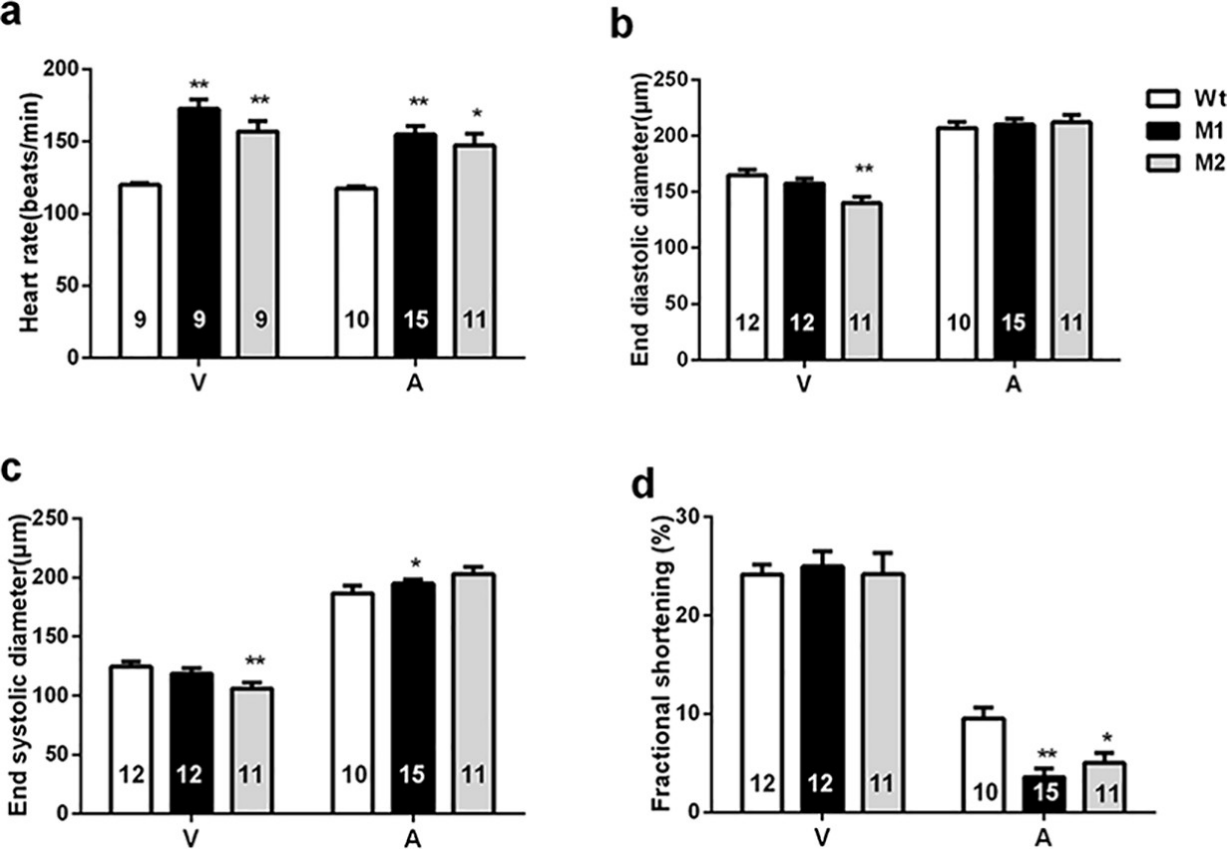
Deletion of *pr72* led to the reduced cardiac function. (a) The heart rate was faster following the deletion of *pr72*. (b) The ventricular end-diastolic diameters were decreased; (c) The ventricular end-systolic diameter was decreased, and the atrial end-diastolic diameter was raised (A, atrium; V, ventricle); (d) Fractional shortening was reduced in the atria. Data are expressed as mean ± SEM. *, *p* < 0.05; **, *p* < 0.01 vs controls, one-way ANOVA followed by Dunnett’s posttest. The numbers of zebrafish are indicated in the columns.

Comparable cardiac phenotypes, particularly the enlargement of ventricular chambers and the reduction of CMs, were noted in these two stable *pr72* KO zebrafish lines (M1 and M2) (Fig 2). Significantly enlarged ventricular chambers and reduced CMs were observed on HE staining sections of adult mutant hearts (Fig 2). And the enlargement of ventricles and the thinning of the atrial wall were started from the embryos; the atria were practically invisible in homozygous mutant 72-hpf-embryos (Fig 2). Furthermore, the functional changes, the remarkably decreased ventricular end-diastolic diameter and end-systolic diameter and atrial fractional shortening, were also observed in 54-hpf-embryos (Fig 3).

### Cardiac ultra-structure was changed in mutants

**Fig 4:**
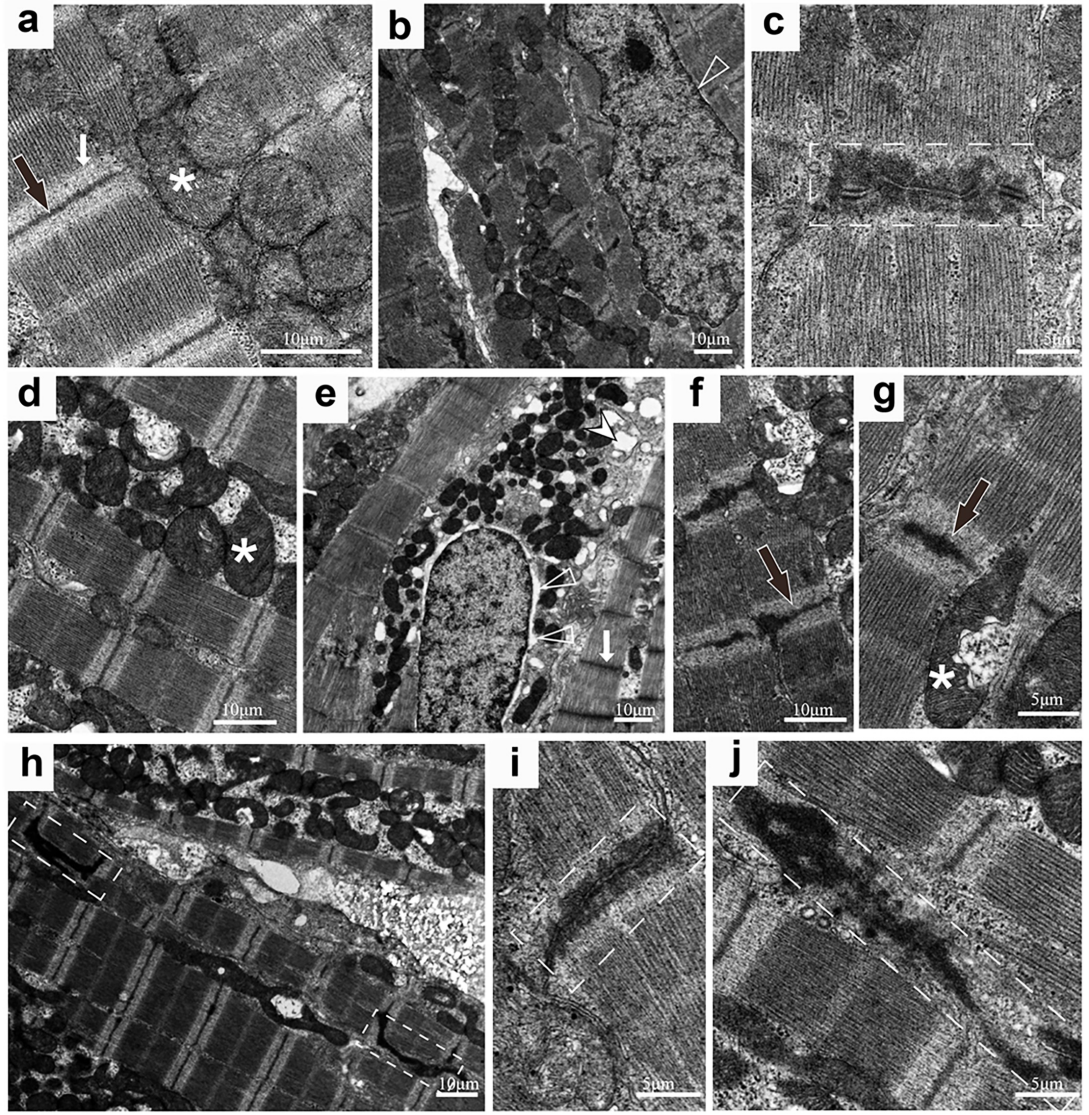
The ultrastructure of zebrafish hearts shown by transmission electron microscopy. In 5 Wt zebrafish, there are normal I bands, Z lines, mitochondria (a) in normal sarcomeres (b), no perinuclear space (b) and a normal ID with four desmosomes (c). In 9 mutated zebrafish, there are swollen mitochondria (d, e, f, g), disappeared I bands, broaden perinuclear spaces and edema (e), thick Z lines (f & g) and vague IDs with paradoxical desmosomes (h, I & j). Scale bars 10μM. (*, mitochondria; white arrows, I band; black arrows, Z line; hollow arrow head, perinuclear spaces; white arrow head, edema; dashed boxes, ID).

The transmission electron microscopy studies uncovered ultra-structural changes in the adult hearts. Being consistent with the myocardium loss, the ultra-structure changes hinted the myocardial degeneration including the swollen mitochondria, the broadened perinuclear space and edema (Fig 4).

Disappearance of the I band, thickening Z lines and vague intercalated disks (IDs) were also observed (Fig 4). Compared with the 100% intact I bands and Z lines in 78 sarcomeres from 5 Wt fish, 15% of the I bands had disappeared and 18% of the Z lines were unevenly thick among 57 sarcomeres from 9 mutant fish. Compared with the 8 IDs with “Z” shapes and clear desmosomes among 89 sarcomeres from 5 Wt fish, 80% of the structure of 12 IDs among 69 sarcomeres from 9 mutant fish were vague and had ambiguous desmosomes (Fig 4).

### Cardiac looping defects could be rescued by *pr72* mRNA

**Fig 5:**
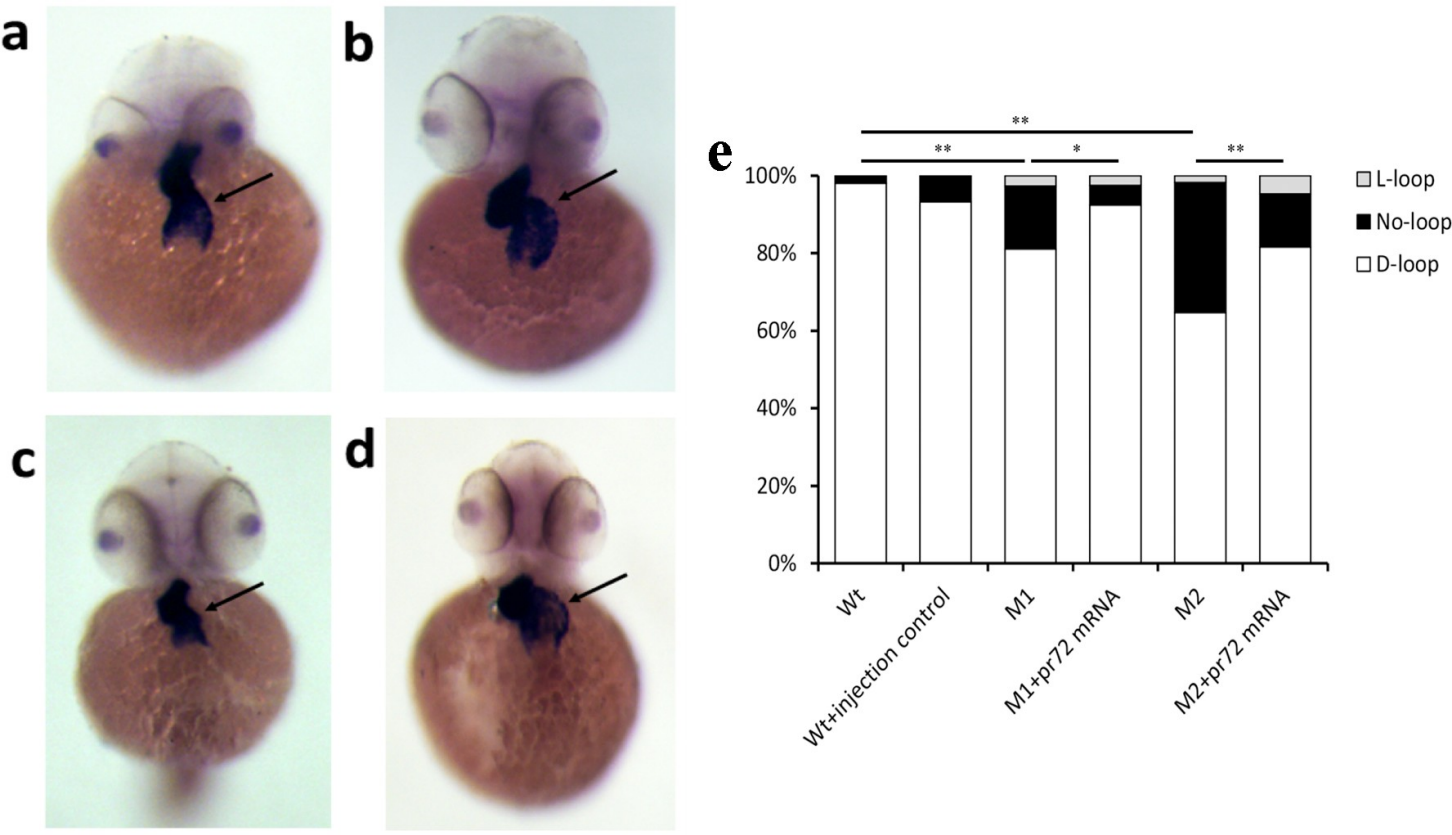
The heart looping before and after rescue. (a) The M1 zebrafish embryo without rescue. (b) The M1 zebrafish with rescue. (c) The M2 zebrafish without rescue. (d) The M2 zebrafish with rescue. (e) The comparison of heart looping in zebrafish embryos at 72 hpf, among WT, WT + injection control, M1, M1 + *pr72* mRNA, M2 and M2 + *pr72* mRNA groups. WT, wild type zebrafish embryos; WT + injection control, wild type zebrafish embryos injected with *pr72* mRNA; M1, mutant zebrafish embryo without rescue; M1+ *pr72* mRNA, the mutant zebrafish with rescue; M2, mutant zebrafish without rescue; M2 + *pr72* mRNA, mutant zebrafish with rescue. The numbers of fish are shown in the columns. Data are expressed as percentages. *, *p* < 0.05; **, *p* < 0.01, unpaired student t test. The experiment was carried out in triplicate.

In 48-hpf zebrafish embryos, the heart underwent a D-looping process, resulting in the atrium on the left side and the ventricle on the right side. In contrast to 99% of Wt zebrafish (n = 103), only 65% of the M2 homozygous mutants (n = 164) and 81% of the M1 homozygous mutants (n = 74) underwent this D-looping process (p<0.05) (Fig 5). The injection of Wt *pr72* mRNA (150 pg) in M2 and M1 homozygous embryos rescued the D-looping to 79% (n = 147), and 92% (n = 159) respectively, supporting the specificity of the looping defect caused by the *pr72* deletion (p<0.05) (Fig 5). However, the looping defect wasn’t completely rescued by Wt *pr72* mRNA. The incomplete rescues might due to that even the injection of Wt *pr72* mRNA in Wt fish could induce slight cardiac looping defects (7%, n = 89) (Fig 5). The representative heart looping images before and after rescues were shown using WISH in Fig 5.

### Pr72 might function through the Wnt/β-catenin pathway

**Fig 6:**
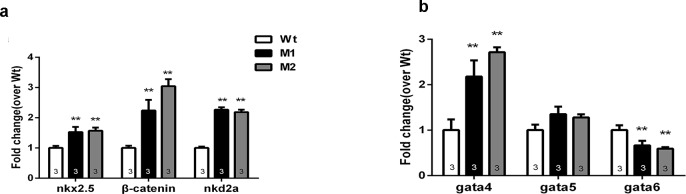
The expression levels of *β-catenin*, *gata4*, *gata5*, *gata*6, *nkd2a* and *nkx2*.*5* in zebrafish embryos at 72 hpf. (a) The expression levels of *β-catenin*, *nkd2a* and *nkx2*.*5*; (b) The expression levels of *gata4*, *gata5*, *gata6*. The mRNA expression was detected in more than 20 zebrafish embryos each time. And the number of replication is shown in the columns. Data are expressed as mean ± SEM. *, *p* < 0.05; **, *p* < 0.01 vs controls, two-way ANOVA followed by Dunnett’s posttest.

PR72 was reported to be a negative regulator of the classical Wnt signaling cascade [14], thus, we compared the expression levels of genes in the Wnt/β-catenin pathway between Wt and mutant, including *β-catenin*, *gata4*, *gata5*, *gata6*, *nkx2*.*5* and *nkd2a*. The expression levels of *β-catenin* and downstream factors, such as *gata4*, *gata6* and *nkx2*.*5* were significantly increased in M1 and M2 fish, which indicated the activation of the Wnt signaling pathway (Fig 6).

## Discussion

To reveal the role of PR72 in heart, the expression pattern of *pr72* in zebrafish was detected and two *pr72* KO zebrafish lines were generated. Pr72 was not maternally deposited in zebrafish differently from in Xenopus, probably due to species difference [14]. However, it expressed from 24-hpf embryos to adult fish, and presented the dominant expression in hearts, which indicated Pr72’s function in zebrafish heart development. Furthermore, we found that the mutant zebrafish exhibited enlarged ventricular chambers and significantly reduced ventricular end-diastolic diameter and atrial fractional shortening. Unfortunately, the cardiac function could be detected only in transparent zebrafish embryos but not in non-transparent adults. But we still observed enlarged ventricular chambers and reduced CMs started from atrium, just like the initial thinning of heart wall was obvious in the embryo-atrium. And the looping defects in mutants derived by *pr72* KO could be rescued by the injection of Wt *pr72* mRNA, which showed that the phenotype was distinctively due to the *pr72* deletion.

This role may possibly be ascribed to a function of *pr72* against the canonical Wnt/ β-catenin (Wnt) signaling pathway. Consistent with this hypothesis, a previous study by Bernards et al. has shown that *pr72* is a negative regulator of the Wnt pathway through combining with Naked cuticle [14]. In our study, the elevation of *β-catenin* and the protein factors downstream of *β-catenin* such as *nkx2*.*5* and *gata4* was observed, which hints the activated Wnt pathway [20–22]. Almost at the same time, two research groups reported that knockdown of another antagonist of Wnt/signaling pathway, Tmem88, activated the canonical Wnt pathway and led to the reduction of myocardium, which had the similar results as ours [23, 24]. We also observed the increase of *nkd2a*, which could be the feedback up-regulation caused by the *pr72* deletion. In addition, we observed *pr72* expression in the branchial arches, while the canonical Wnt pathway has been found to regulate craniofacial patterning and morphogenesis within the brachial arches, so Pr72 might be required for development of branchial arches as a negative regulator of Wnt pathway[25].

Using transmission electron microscopy, we observed that the I bands disappeared, the thick Z lines became uneven and intercalated disks (IDs) grew vague with a decreased number of small desmosomes in the sarcomeres of *pr72* KO fish. These structural changes in sarcomeres might possibly be a direct consequence of the defective dephosphorylation process of the thin filament proteins. PP2A has been shown to play a critical role in the dephosphorylation of thin filament proteins, including Actin, Tropomyosin, and the Troponin complex (Troponin C, I and T), which regulates myofilament contractility [6, 26]. For example, Troponin I dephosphorylation by PP2A increases the Ca^2+^ sensitivity of the myofilament. The disappeared I band and increased heart rate in the *pr72* KO fish, might due to the abnormal dephosphorylation of the thin filament proteins by affecting Ca^2+^ sensitivity of myofilament. Compared with the widely expressed PP2A, the surprising diversity in the expression and distribution of the regulatory B subunit may be related to the substrate specificity and subcellular localization of PP2A [27]. Being consistent with an important function of PP2A in regulating myofilaments, the regulator subunit, PPP2R3A has been found to locate in the Z-lines of the CM [2]. Thus, we hypothesized the sick and uneven Z band could be the results of *pr72* deletion.

In our previous study, deletion of *pr130* in zebrafish resulted in cardiac developmental defects, including cardiac looping defects, reduced cardiomyocytes, disturbance of cardiac ultra-structure and decreased cardiac function [15]. Here, we show that *pr72*, which has an N terminus that differ from *pr130* but sharing the same C terminus, exhibited the similar phenotype in heart. But the changes of cardiac ultra-structure in *pr72* and *pr130* are not identical. The cause accounting for the differential phenotype is unknown, or probably due to the difference of the N terminus.

One of the limitations of TALEN-based insertion or deletion of the target site is that this process may introduce off-target insertions or deletions. To eliminate the potential for off target effects, we used the siblings of the mutant zebrafish (Wt offspring) as the controls, which had the same parents and genetic background with mutants. And we got the replication of results using the second independent method using morpoline (S1 Text and S1 Fig).

In conclusion, phenotypic studies in zebrafish uncovered important functions of the regulator subunit PR72 in cardiac development and determined that its function cannot be fully compensated by other regulator subunits.

## Supporting information

S1 Table Primer sequences. (DOCX)

S1 Text Supplementary methods and results. (DOCX)

S1 Fig Morpholino knockdown of *pr72* in zebrafish causes cardiac phenotypes. (a) Bright field images of zebrafish embryos at 72hpf. Wt, untreated control group; STD-MO, standard control morpholino group; ATG-MO, translation initiation blocking morpholino group. Arrow marks pericardial effusion. (b) Expressions of cmlc2: EGFP in the heart of cmlc2: EGFP transgenic zebrafish embryos at 72hpf. Abnormal enlarged atrium is indicated by red arrow in ATG-MO group. (c) Expression of *cmlc2* in the ATG-MO, STD-MO embryos and untreated control embryos at 24 and 72 hpf. Red arrow shows abnormal *cmlc2* expression in the atrium, which represents enlarged atrium.
(TIF)

## References

[1] Hood AR, Ai X, Pogwizd SM. Regulation of cardiac gap junctions by protein phosphatases. Journal of molecular and cellular cardiology. 2017;107 (Supplement C):52–7. 10.1016/j.yjmcc.2017.05.002. 28478048 PMC6423971

[2] DeGrande ST, Little SC, Nixon DJ, Wright P, Snyder J, Dun W, et al Molecular mechanisms underlying cardiac protein phosphatase 2A regulation in heart. The Journal of biological chemistry. 2013;288(2):1032–46. Epub 2012/12/04. 10.1074/jbc.M112.426957 ; PubMed Central PMCID: PMCPMC3542989. 23204520 10.1074/jbc.M112.426957 PMC3542989

[3] Shan J, Kushnir A, Betzenhauser MJ, Reiken S, Li J, Lehnart SE, et al Phosphorylation of the ryanodine receptor mediates the cardiac fight or flight response in mice. The Journal of clinical investigation. 2010;120(12):4388–98. 10.1172/JCI32726 ; PubMed Central PMCID: PMC2993575. 21099118 10.1172/JCI32726 PMC2993575

[4] Shi Y. Serine/threonine phosphatases: mechanism through structure. Cell. 2009;139(3):468–84. Epub 2009/11/03. 10.1016/j.cell.2009.10.006 . 19879837 10.1016/j.cell.2009.10.006

[5] Ranieri A, Kemp E, Burgoyne JR, Avkiran M. beta-Adrenergic regulation of cardiac type 2A protein phosphatase through phosphorylation of regulatory subunit B56delta at S573. Journal of molecular and cellular cardiology. 2018;115:20–31. Epub 2018/01/03. 10.1016/j.yjmcc.2017.12.016 ; PubMed Central PMCID: PMCPMC5823843. 29294329 10.1016/j.yjmcc.2017.12.016 PMC5823843

[6] Eichhorn PJ, Creyghton MP, Bernards R. Protein phosphatase 2A regulatory subunits and cancer. Biochimica et biophysica acta. 2009;1795(1):1–15. 10.1016/j.bbcan.2008.05.005 . 18588945 10.1016/j.bbcan.2008.05.005

[7] Lubbers ER, Mohler PJ. Roles and regulation of protein phosphatase 2A (PP2A) in the heart. Journal of molecular and cellular cardiology. 2016;101 (Supplement C):127–33. 10.1016/j.yjmcc.2016.11.003. 27832939 PMC5939568

[8] Li L, Fang C, Xu D, Xu Y, Fu H, Li J. Cardiomyocyte specific deletion of PP2A causes cardiac hypertrophy. American journal of translational research. 2016;8(4):1769–79. Epub 2016/05/18. ; PubMed Central PMCID: PMCPMC4859906. 27186301 PMC4859906

[9] Heijman J, Ghezelbash S, Wehrens XHT, Dobrev D. Serine/Threonine Phosphatases in Atrial Fibrillation. Journal of molecular and cellular cardiology. 2017;103:110–20. 10.1016/j.yjmcc.2016.12.009. 28077320 PMC5346472

[10] Gergs U, Boknik P, Buchwalow I, Fabritz L, Matus M, Justus I, et al Overexpression of the catalytic subunit of protein phosphatase 2A impairs cardiac function. The Journal of biological chemistry. 2004;279(39):40827–34. 10.1074/jbc.M405770200 . 15247211 10.1074/jbc.M405770200

[11] Brewis N, Ohst K, Fields K, Rapacciuolo A, Chou D, Bloor C, et al Dilated cardiomyopathy in transgenic mice expressing a mutant A subunit of protein phosphatase 2A. American journal of physiology Heart and circulatory physiology. 2000;279(3):H1307–18. Epub 2000/09/20. 10.1152/ajpheart.2000.279.3.H1307 . 10993798 10.1152/ajpheart.2000.279.3.H1307

[12] Varadkar P, Despres D, Kraman M, Lozier J, Phadke A, Nagaraju K, et al The protein phosphatase 2A B56gamma regulatory subunit is required for heart development. Developmental dynamics: an official publication of the American Association of Anatomists. 2014;243(6):778–90. Epub 2014/01/16. 10.1002/dvdy.24111 . 24425002 10.1002/dvdy.24111

[13] Glaser D, Müller FU, Stümpel F, Boknik P, Kirchhefer U. Abstract 20080: Deletion Of B56α, a Regulatory Subunit of Protein Phosphatase 2A, is Associated With Improved Cardiac Performance. Circulation. 2017;136:A20080–A.

[14] Creyghton MP, Roel G, Eichhorn PJ, Hijmans EM, Maurer I, Destree O, et al PR72, a novel regulator of Wnt signaling required for Naked cuticle function. Genes & development. 2005;19(3):376–86. Epub 2005/02/03. 10.1101/gad.328905 ; PubMed Central PMCID: PMCPMC546515. 15687260 10.1101/gad.328905 PMC546515

[15] Yang J, Li Z, Gan X, Zhai G, Gao J, Xiong C, et al Deletion of Pr130 Interrupts Cardiac Development in Zebrafish. International journal of molecular sciences. 2016;17(11). Epub 2016/11/16. 10.3390/ijms17111746 ; PubMed Central PMCID: PMCPMC5133774. 27845735 10.3390/ijms17111746 PMC5133774

[16] Chen X, Lou Q, He J, Yin Z. Role of zebrafish lbx2 in embryonic lateral line development. PloS one. 2011;6(12):e29515 10.1371/journal.pone.0029515 ; PubMed Central PMCID: PMC3245281. 22216300 10.1371/journal.pone.0029515 PMC3245281

[17] Sander JD, Cade L, Khayter C, Reyon D, Peterson RT, Joung JK, et al Targeted gene disruption in somatic zebrafish cells using engineered TALENs. Nature biotechnology. 2011;29(8):697–8. 10.1038/nbt.1934 ; PubMed Central PMCID: PMC3154023. 21822241 10.1038/nbt.1934 PMC3154023

[18] Cade L, Reyon D, Hwang WY, Tsai SQ, Patel S, Khayter C, et al Highly efficient generation of heritable zebrafish gene mutations using homo- and heterodimeric TALENs. Nucleic acids research. 2012;40(16):8001–10. 10.1093/nar/gks518 ; PubMed Central PMCID: PMC3439908. 22684503 10.1093/nar/gks518 PMC3439908

[19] Zhang M, Mal N, Kiedrowski M, Chacko M, Askari AT, Popovic ZB, et al SDF-1 expression by mesenchymal stem cells results in trophic support of cardiac myocytes after myocardial infarction. FASEB journal: official publication of the Federation of American Societies for Experimental Biology. 2007;21(12):3197–207. 10.1096/fj.06-6558com . 17496162 10.1096/fj.06-6558com

[20] Durak O, de Anda FC, Singh KK, Leussis MP, Petryshen TL, Sklar P, et al Ankyrin-G regulates neurogenesis and Wnt signaling by altering the subcellular localization of beta-catenin. Molecular psychiatry. 2014 10.1038/mp.2014.42 ; PubMed Central PMCID: PMC4231016. 24821222 10.1038/mp.2014.42 PMC4231016

[21] Lin X, Xu X. Distinct functions of Wnt/beta-catenin signaling in KV development and cardiac asymmetry. Development. 2009;136(2):207–17. 10.1242/dev.029561 . 19103803 10.1242/dev.029561

[22] Martin J, Afouda BA, Hoppler S. Wnt/beta-catenin signalling regulates cardiomyogenesis via GATA transcription factors. Journal of anatomy. 2010;216(1):92–107. 10.1111/j.1469-7580.2009.01171.x ; PubMed Central PMCID: PMC2807978. 20402826 10.1111/j.1469-7580.2009.01171.x PMC2807978

[23] Novikov N, Evans T. Tmem88a mediates GATA-dependent specification of cardiomyocyte progenitors by restricting WNT signaling. Development. 2013;140(18):3787–98. 10.1242/dev.093567 ; PubMed Central PMCID: PMC3754477. 23903195 10.1242/dev.093567 PMC3754477

[24] Palpant NJ, Pabon L, Rabinowitz JS, Hadland BK, Stoick-Cooper CL, Paige SL, et al Transmembrane protein 88: a Wnt regulatory protein that specifies cardiomyocyte development. Development. 2013;140(18):3799–808. 10.1242/dev.094789 ; PubMed Central PMCID: PMC3754478. 23924634 10.1242/dev.094789 PMC3754478

[25] Jin YR, Turcotte TJ, Crocker AL, Han XH, Yoon JK. The canonical Wnt signaling activator, R-spondin2, regulates craniofacial patterning and morphogenesis within the branchial arch through ectodermal-mesenchymal interaction. Dev Biol. 2011;352(1):1–13. 10.1016/j.ydbio.2011.01.004 ; PubMed Central PMCID: PMCPMC3089906. 21237142 10.1016/j.ydbio.2011.01.004 PMC3089906

[26] Yin X, Cuello F, Mayr U, Hao Z, Hornshaw M, Ehler E, et al Proteomics analysis of the cardiac myofilament subproteome reveals dynamic alterations in phosphatase subunit distribution. Molecular & cellular proteomics: MCP. 2010;9(3):497–509. Epub 2009/12/29. 10.1074/mcp.M900275-MCP200 ; PubMed Central PMCID: PMCPMC2849712. 20037178 10.1074/mcp.M900275-MCP200 PMC2849712

[27] Perrotti D, Neviani P. Protein phosphatase 2A: a target for anticancer therapy. The lancet oncology. 2013;14(6):e229–38. 10.1016/S1470-2045(12)70558-2 ; PubMed Central PMCID: PMC3913484. 23639323 10.1016/S1470-2045(12)70558-2 PMC3913484

