# Supplementary material for: Deletion of Pr72 causes cardiac developmental defects in Zebrafish
Source: PLoS One. 2018 Nov 27;13(11):e0206883. doi: 10.1371/journal.pone.0206883 (PMC6258505; doi:10.1371/journal.pone.0206883)
Supplement: S1 Table — (DOCX) [file pone.0206883.s001.docx]

**S Table 1. Primer sequences.**

| **Usages** | **Genes** | **Forward and reverse primers（ 5'→ 3'）** | **Product length (bp)** |
| --- | --- | --- | --- |
| RT-qPCR | *pr72*-F | GACCCACCTTGACGGAAGTCCCTGAG | 246 |
|  | *pr72*-R | GGCGTGGTGGTTCTCCATTCAACATCACTG |  |
| Probe synthesis | *pr72*-F | CCCGTTGGCTATGGACTTGTGTTG |  |
|  | *pr72*-R | CTGAGGACGGTGCAGGTGATAGT |  |
|  | *cmlc2*-F | CAGACCAACAGCAAAGCAG |  |
|  | *cmlc2*-R | CTGAGAGCAACTGAGTATGAAG |  |
| qPCR | *pr72*-F | GGGAAGAATGAGCTATGCAGAT | 171 |
|  | *pr72*-R | GCTTCCATCCGTTCACACT |  |
|  | *GAPDH*-F | GAAGGTGAAGGTCGGAGTC | 226 |
|  | *GAPDH*-R | GAAGATGGTGATGGGATTTC |  |
|  | *β-catenin*-F | GAGAGGAAGCCCCTGGCAAG | 203 |
|  | *β-catenin*-R | GGCCATCACCACGTCCTCAG |  |
|  | *nkd2a*-F | GCCTCCAGAGAAAGCTAAG | 239 |
|  | *nkd2a*-R | CCACCACATCATAGATAGATAGTGTGC |  |
|  | *nkx2.5*-F | GCGAAGACCTTCCAGGAGGAC | 250 |
|  | *nkx2.5*-R | GCCTGAGAGAAGAGAACTCGAG |  |
|  | *gata4*-F | CTATGTGAGCCCTAATATCGGAGC | 240 |
|  | *gata4*-R | CCGTTCATCTTATGGTAGAGTCCAC |  |
|  | *gata5*-F | AGATGGCAACCTCATGGACG | 229 |
|  | *gata5*-R | CCGTTCATCTTGTGGTAAAGGC |  |
|  | *gata6*-F | TATGTCACACCGCAGCTCTC | 264 |
|  | *gata6*-R | TAATGGTCGGCTGAGTCCATTC |  |
|  | *β-actin*-F | GCCGTGACCTGACTGACTACCT | 273 |
|  | *β-actin-R* | CGCAAGATTCCATACCCAAGA |  |
