# Supplementary material for: Deletion of Pr72 causes cardiac developmental defects in Zebrafish
Source: PLoS One. 2018 Nov 27;13(11):e0206883. doi: 10.1371/journal.pone.0206883 (PMC6258505; doi:10.1371/journal.pone.0206883)
Supplement: S1 Text — (DOCX) [file pone.0206883.s002.docx]

**S1 Text. Supplementary methods and results**

The replication of results using morpholinos has been carried on. Morpholino oligonucleotide (MO) directed against the translation initiation codon (ATG-MO) and standard control MO (STD-MO) were purchased from Gene Tools, LLC (Philomath, OR). The MOs were microinjected into 1- to 2-cell stage zebrafish embryos. Zebraﬁsh embryos of controls and *pr72* morphants were allowed to develop until 72 hpf for the evaluation of general development process of the heart. The morphological features of zebrafish heart were directly observed in cmlc2: EGFP transgenic zebrafish or visualized with the expression patterns of *cmlc2* via WISH.

The cardiac phenotypes were analyzed in Wt zebrafish injected with ATG-MO or STD-MO. Compared with those of the STD-MO morphants, large pericardial effusions were observed in ATG-MO morphants (S1 Fig). Utilizing cmlc2: EGFP transgenic line and WISH analyses of endogenous *cmlc2* expression, atrial enlargement characterized with *cmlc2* expression patterns, which labeled cardiac myocytes in both ventricle and atrium, were observed in ATG-MO injected embryos (S1 Fig).
