# Supplementary figures and images for: Deletion of Pr72 causes cardiac developmental defects in Zebrafish
Source: PLoS One. 2018 Nov 27;13(11):e0206883. doi: 10.1371/journal.pone.0206883 (PMC6258505; doi:10.1371/journal.pone.0206883)

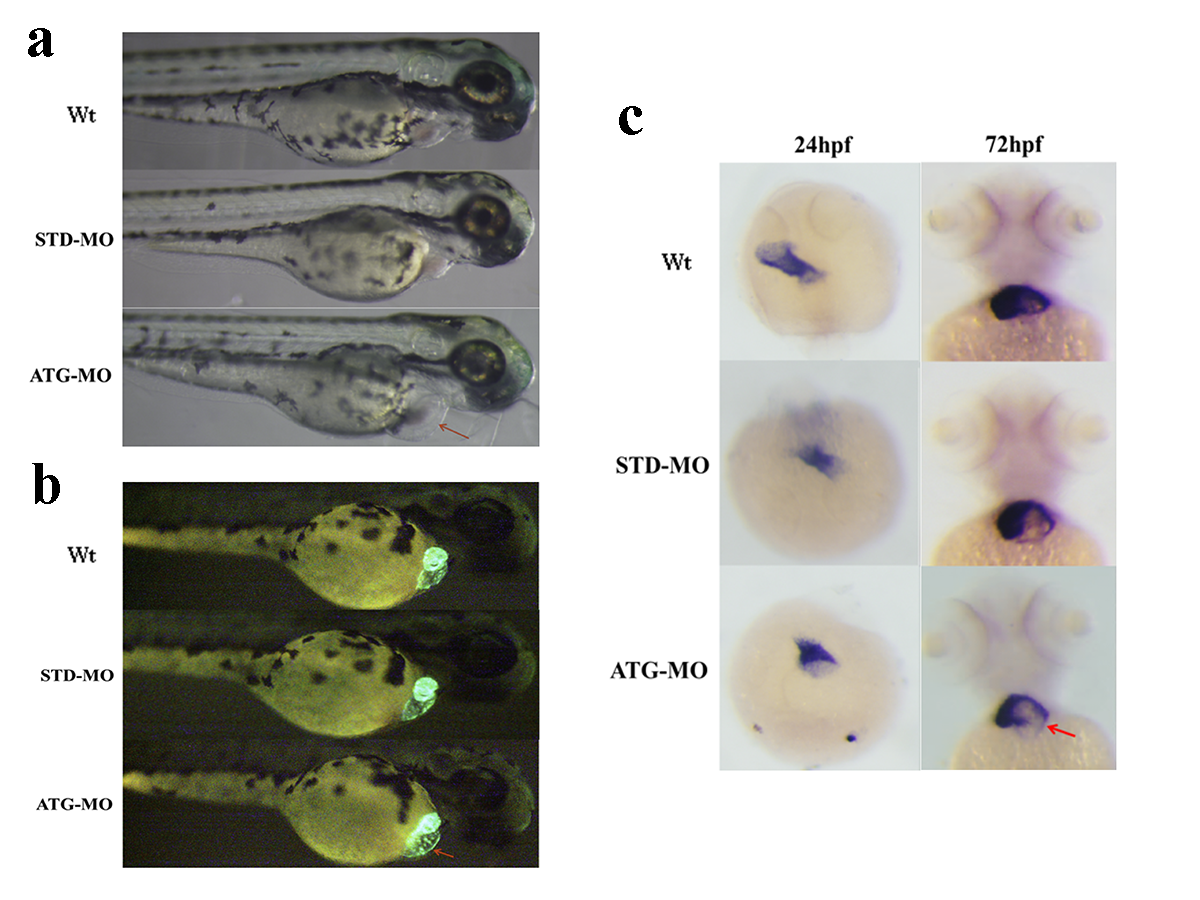

Supplement: S1 Fig — (a) Bright field images of zebrafish embryos at 72hpf. Wt, untreated control group; STD-MO, standard control morpholino group; ATG-MO, translation initiation blocking morpholino group. Arrow marks pericardial effusion. (b) Expressions of cmlc2: EGFP in the heart of cmlc2: EGFP transgenic zebrafish embryos at 72hpf. Abnormal enlarged atrium is indicated by red arrow in ATG-MO group. (c) Expression of cmlc2 in the ATG-MO, STD-MO embryos and untreated control embryos at 24 and 72 hpf. Red arrow shows abnormal cmlc2 expression in the atrium, which represents enlarged atrium. (TIF) [file pone.0206883.s003.tif]
